# *Cadosinopsis rehakovii* sp. nov., a new calcareous dinocyst from the Jurassic-Cretaceous transitional interval of the Western Tethys

**DOI:** 10.1371/journal.pone.0249690

**Published:** 2021-05-05

**Authors:** Agnieszka Ciurej, Marta Bąk

**Affiliations:** 1 Geology Department, Institute of Geography, Pedagogical University of Krakow, Kraków, Poland; 2 Faculty of Geology, Geophysics, and Environmental Protection, AGH University of Science and Technology, Kraków, Poland; Università degli Studi di Torino, ITALY

## Abstract

Variegated limestones, a transitional series between red, Upper Jurassic radiolarite and whitish, Lower Cretaceous Maiolica limestone in the Pieniny Klippen Belt deposits in the Polish part of the Western Carpathians, have yielded rich microfossil assemblages with common calcareous dinoflagellate resting stages, hereafter, dinocysts. We found an undescribed dinocyst species in red-greenish limestone of a deep water, pelagic habitat in the Branisko succession of the Pieniny Klippen Basin and named it *Cadosinopsis rehakovii* sp. nov. The new species has a spherical to oval calcareous test ranging from 34 to 59 μm in length and 30 to 50 μm in width, with two layered wall. The inner layer is built of coarse–thick, plate-shaped calcite crystals and is white in transmitted light. The outer layer is built of fibrous crystals, vitreous (transparent) in transmitted light. The aperture is wide and seen only in the inner layer. We compared the detailed morphological characteristics of the new species with another species from the same genus in the Jurassic and Cretaceous Tethyan deposits. Specimens have been measured, grouped and interpreted using cluster analysis, principal component analysis (PCA) and canonical variate analysis (CVA). Among them, the new species shows a resemblance in cross-section to other species of *Cadosinopsis*, *C*. *nowaki* Borza, 1984, and *C*. *andrusovi* Scheibner 1967, previously described in literature. However, the two species are easily distinguishable by some features. *Cadosinopsis*. *nowaki* is bigger in size (length from 50 to76 μm and width from 43 to 67 μm), its inner layer is thicker and consists of vitreous-sparite calcite, and it has less centrically located chamber. *C*. *andrusovi* is much bigger in size as its length ranges from 68 to 108 μm and width ranges from 60 to 80 μm, and the cyst is more oval and its chamber less spherical. The new species is the third *Cadosinopsis* species described in the Tethyan realm and about two hundred and sixty-first fossil species (morphotype) described in the world so far.

## Introduction

Single-celled spherical to ovoid, calcareous microfossils known in the fossil record from the Paleozoic to the Cenozoic have been usually referred to as “calcispheres” [1]. Many of the so-called "calcispheres" has been classified into different taxonomic groups of protists and plants [2] such as foraminifers, calcareous algal spores or spores of dasyclad algae. Despite many previous investigations, the biological affinities of many “calcispheres” remain unknown. For these forms Versteegh et al. [3] proposed a new incertae sedis group of the Calcitarcha that includes all calcareous microfossils with a central cavity and currently lacking taxonomic allocation.

Calcareous dinoflagellates (or calcareous cyst-producing dinoflagellates, or calcareous dinocysts) are thought to be a monophyletic group belonging to the order Peridiniales Dinoflagellata group. They are photosyntethic planktonic organisms (e.g. [4–6]). About 260 fossil species (morphotypes) [7] and about 30 extant species of calcareous dinoflagellates [8–12] have been described so far. The oldest known examples are from the upper Triassic [13]. They form calcareous cysts (calcareous outer skeleton), that ranges in size from about 7 to 100 μm during their life cycle (e.g. [12, 14–16]). Two functional types of calcareous cysts have been recognized so far [4, 17, 18] as resting and vegetative.

Nowadays the taxonomy of calcareous dinoflagellates is based on a system dealing with the recent and fossil characteristics (see [19] and literature therein). The most useful characters are: (a) wall structures (including crystallographic orientation, crystal morphology and number of layers in the wall), (b) shape and size of the cysts, (c) optical properties of walls of cysts, (d) type of archeopyle/operculum (size, shape and plate numbers that constitute arheophyle/operculum), (e) the character and orientation of the cyst-theca relations (e.g., tabulation and paratabulation pattern), and (f) recently also molecular records (e.g. [3, 19–27]). The taxonomy of the fossil specimens can be problematic. Diagenetic problems can preclude the valid identification of calcareous dinocysts at the species level. The useful characteristics can be overprinted during diagenesis. The most important problems include: (1) cyst wall can be modified by secondary crystal growth, and the crystallographic orientation of the calcite crystals can be obliterated, (2) shape, size, outer and inner cyst morphology, details of the surface and wall structures can be modified through diagenetic process.

Calcareous dinocysts and Calcitarcha assemblages are relatively common within the Upper Jurassic through Lower Cretaceous deposits from the Tethyan realm of the Carpathians (mostly Tithonian–Albian). They are especially numerous in Western Carpathian settings (e.g., [28–47]).

The genus *Cadosinopsis* has been proved to represent dinoflagellate calcareous cysts in resting, reproductive or coccoid stages of their life cycle [48] belonging to the Family Thoracosphaeraceae [19]. It was first introduced by Scheibner [49] for unilocular microorganisms observed in thin sections of the rocks. These forms were oval to sub-oval in shape and possess a two-layered calcite wall. In the original description, the inner layer of the wall was coarser-grained, porcellaneous with one opening, while the outer layer which possesses several pores, was built of radially arranged fine lamellae of calcite. The first species of this genus—*Cadosinopsis andrusovi* Scheibner 1967 was found and described from marly sequence with abundant Santonian globotruncanids [49]. The second species of the genus *Cadosinopsis* named *C*. *nowaki* was described by Borza [50] from the Hauterivian marly limestone of the Križna nappe and Manin unit as well as conglomerates of Santonian-Campanian age in the Klape unit of the Klippen Belt in the Western Carpathians [50]. *Cadosinopsis nowaki* was also found in localities in the Polish part of the Outer Carpathians (e.g., [44, 45]). Recently, Skupien and Dupovcova [51] found *C*. *nowaki* in the upper Tithonian deposits in the Western Carpathians, in Czech Republic.

Ivanova [52] widened the paleogeographic distribution of genus *Cadosinopsis* as she described species C. *nowaki* from new localities in the West Forebalkan and West Balkan from Bulgaria. This author also proposed a new Cadosinopsis nowaki Zone, the base of which is defined by the earliest appearance of *C*. *nowaki* in the uppermost part of the Valanginian.

The present paper evaluates the diversification of *Cadosinopsis* in the Carpathians in comparison with representatives described from Tethyan low latitude localities. The new species *Cadosinopsis rehakovii* sp. nov. is also described. The taxon discussed herein occurs in a rich and diverse Lower Tithonian assemblage within the Branisko succession of the Pieniny Klippen Belt (Western Carpathians). All the presented cysts are only slightly affected by diagenetic recrystallization. A qualitative approach to systematics has been employed, combined to detailed measurements and statistical analyses.

## Material and methods

### Location of samples investigated

**Fig 1:**
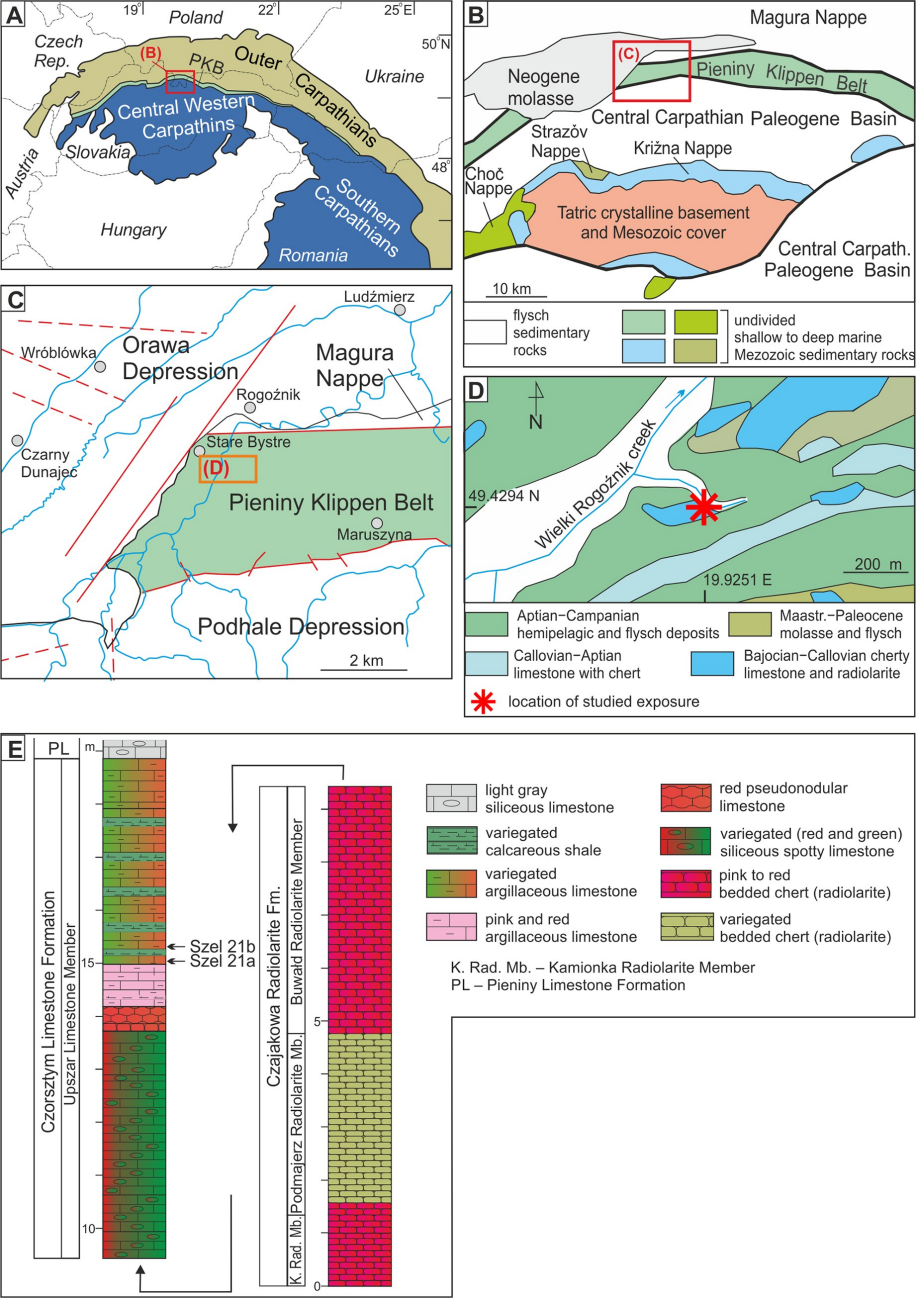
Position of the studied section of the Szeligowy Creek in the Pieniny Klippen Belt. A—The Carpathians as the Alpine orogen. (B-D) Simplified geological maps of the Pieniny Klippen Belt (B–modified after [54]; C–modified after [55], D–simplified after [56]) with the detailed location of the studied area section of Szeligowy Creek, E–Lithological column at Szeligowy Creek as logged in 1998.

The material studied includes 33 samples from the Szeligowy Creek section located in the Polish part of the Pieniny Klippen Belt (Fig 1A). This structural unit represents a zone of strongly deformed Mesozoic and Paleogene sedimentary rocks which separates now the Inner and Outer Carpathians (e.g., [53]). During the Late Jurassic–Early Cretaceous time interval the Pieniny Klippen Basin consisted of several sub-basins with prevalent pelagic, deep-water sedimentation. The Branisko Succession sediments were deposited within the lower bathyal zone (e.g., [53]).

The study area is located in the southern part of Poland in the Nowy Targ Depression. There, the Pieniny Klippen Basin is compressed to a width of several hundred meters to a few kilometers forming a narrow strongly folded and tectonized zone, locally outcropping. One of the outcrop is located in the Szeligowy creek which is the right tributary of the Wielki Rogoźnik river. The investigated section is located in an undercutting of the Quaternary terrace, 150 m up stream from the mouth of the creek (Fig 1B). Deposits belonging to the Branisko Succession represent the uppermost part of the red radiolarite sequence which passes into the Maiolica-type cherty limestone.

According to the stratigraphical division of the Pieniny Klippen Basin deposits [53, 57], red radiolarites belong to the Buwałd Radiolarite Member of the Czajakowa Radiolarite Formation and the Maiolica-type limestone represents the Pieniny Limestone Formation. These units are subdivided by reddish to greenish limestone with intercalation of thin radiolarite layers that are classified to the Upszar Limestone Member of the Czorsztyn Limestone Formation which usually consists of red nodular limestone in other parts of the Pieniny Klippen Basin (Fig 1E).

**Fig 2:**
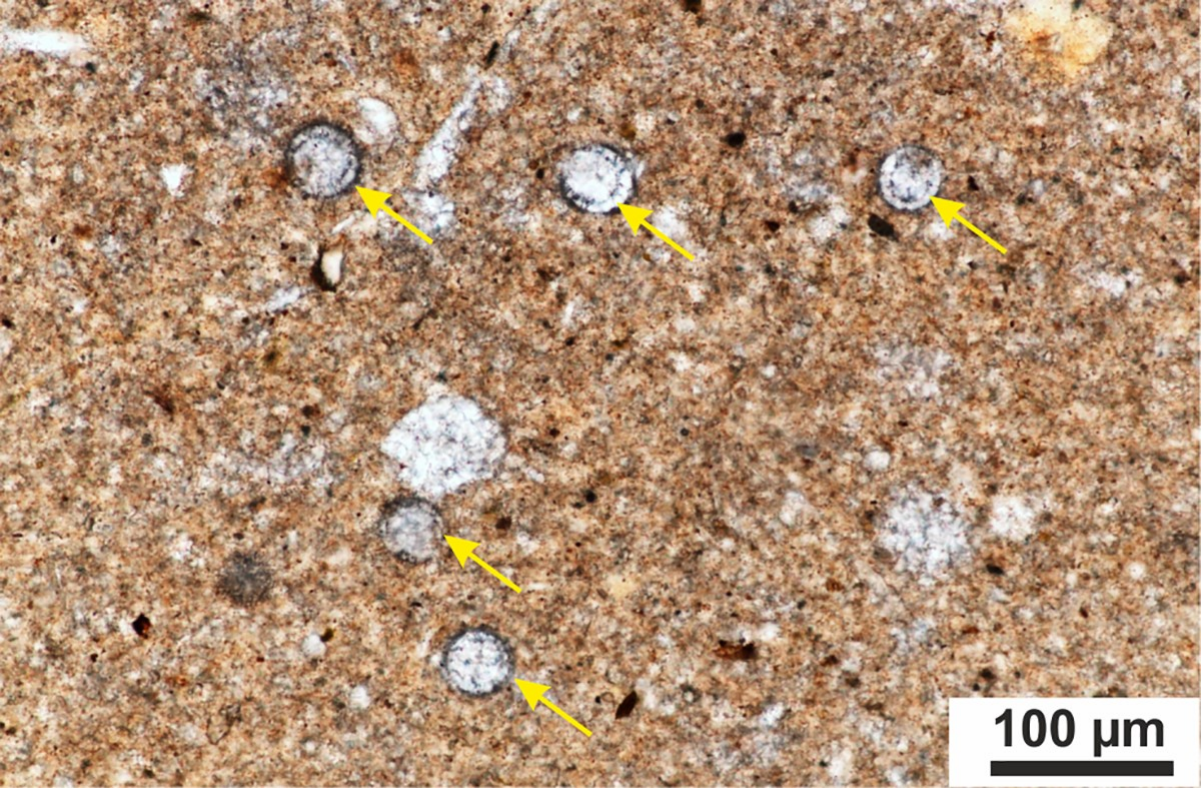
Microscopic view of wackestone rich in *Cadosinopsis rehakovii* sp. **nov. observed in longitudinal and transversal sections (arrows).** Thin section 1N, No. Szel 21b.

Calcareous dinocysts were studied in samples collected every 20–40 cm of the studied section. The newly defined *Cadosinopsis rehakovii sp*. *nov*. has been found in samples Szel 21a and Szel 21b which were taken from the first layer with greenish intercalation within the upper part of the Upszar Limestone Member (Fig 1E). Sample Szel 21a contains 66 specimens, where samples Szel 21b contains an abundant set of *Cadosinopsis rehakovii* sp. nov. with 165 recognized specimens (Fig 2).

The digging and site access permits of these rock samples are not required for this study. There are no legal or ethical restrictions being placed upon the data.

### Preparation of samples, observation methods and storage

The material used in this study was originally collected for radiolarians by Marta Bąk (AGH University of Science and Technology, Kraków, Poland). Calcareous dinocysts were observed in thin sections of the rocks of 3x5 cm size. Thin sections were arbitrarily divided into intervals that were characterized by different percentages of calcareous dinocysts. The thin sections with the newly defined *Cadosinopsis rehakovii sp*. *nov*. have been divided as following: thin section (sample) with No. Szel 21a into 4 intervals and thin section (sample) No. Szel 21b into 5 intervals.

The observations were made under a Nikon Eclipse LV100N POL polarizing optical microscope with a digital camera and NIS-Elements BR software (Department of Geology, Pedagogical University of Krakow).

In order to obtain the optimal image of the specimens, different parameters of the microscope instrumentation settings were used, including various light parameters: (a) transmitted light (plane polarized light), (b) crossed polars, (c) lowered distance of condenser lens from stage with sample and (d) open or semi closed aperture diaphragm. Aperture diaphragm adjustment and proper focusing of the condenser (with regard to height of the objective) make it possible to control the depth of field, improving the image quality and the specific details of the observed specimens. The semi-closed aperture gives good contrast and relief (Becke-line methods) which allows for observation of the wall structure of the test of calcareous dinocysts, the mineralogical phases, and possibility improves the visibility of calcareous cysts within the calcareous host material. If the diaphragm was closed down too much, the image of calcareous dinocysts was "grainy" with much less resolution. Whereas in some cases if the aperture is wide open, the image would appear without relief and with no contrast.

Calcareous dinocysts were also observed in the rock chips under scanning electron microscopy (SEM) at HITACHI 3–4700 housed at the Institute of Geological Sciences of the Jagiellonian University, Kraków, Poland. The broken surface (without any chemical treatment) of the rock chips, in size 2x3 cm, was coated by gold and observed under secondary electron (SE) mode, with acceleration voltage was set at 20 keV on a high vacuum, and work distance was set approximately 13.0 mm (12,4 mm to 14.4 mm).

The holotype and paratypes (located within thin sections no: Szel 21a and Szel 21b) are deposited in the collections of the European Micropaleontological Reference Centre, Address: Micropress Europe al. Mickiewicza 30; 30–059 Krakow, Poland, email: info@micropresseurope.eu and housed in Cabinet 7, drawer 11A. Collection reference is EMRC 7/11A.

Institutional abbreviation.—

EMRC—European Micropaleontological Reference Centre

-

Other abbreviations: TS–thin section, 1N - transmitted light, XN–crossed polars, OA—open aperture diaphragm, SCA–semi- closed aperture diaphragm, LDC—Lowered distance of condenser lens from stage with sample SEM—scanning electron microscopy

### Statistical analysis

From 231 specimens of *Cadosinopsis* found in samples Szel 21a and Szel 21b, 82 of the best preserved cysts were measured and grouped by statistical methods. The specimens illustrated and described as *Cadosinopsis nowaki* by Borza [50] and specimens described as *Cadosinopsis andrusovi* by Scheibner [49] were also measured included in the dataset and compared with the newly described holotype and paratypes of *Cadosinopsis rehakovii* sp. nov. The specimen parameters used in the calculations are listed in Tables 1 and 2. A combination of principal component analysis (PCA), canonical variates analysis (CVA) and cluster analysis were used for calculations. Statistical analyses were carried out on the original specimen dimensions (Tables 1 and 2) using the software package PAST-Palaeontological Statistics, ver. 0.97, written by Ryan et al. [58]. Explanations of statistical techniques implemented there are presented in Harper [59] and Hammer et al. [60]. Cluster analysis was employed to find hierarchical groupings in the multivariate dataset. The dendrograms derived from Ward’s method (with Euclidean distance) and the unweighted pair–group average (computed separately with Chord distance and Morisita’s index) were compared. As the groupings were effectively the same after using these methods, only one dendrogram, constructed using Ward’s method is presented.

**Table 1:** Dimensions of the specimens of *Cadosinopsis rehakovii* sp. nov., specimens 1–81 measured from thin section no. Szel 21b and specimen 82 from thin section no. Szel 21a.

Specimens number	Interval number	L [μm]	W [μm]	TO [μm]	TI [μm]	L/W	Remarks	Repository number	Figure
1	1	52	45	4	5	1.16			Pl.2.C
2	1	45	42	5	5	1.07			
3	1	50	46	3	10	1.09			
4	1	34	30	2.5	3	1.13			
5	1	50	44	4	5	1.14			
6	1	52	48	4	6	1.08			
7	1	42	40	2.5	5	1.05			
8	1	43	40	2	5	1.08			
9	1	51	47	4.5	6	1.09			
10	1	51	46	4	8	1.11			Pl.3.A
11	1	43	42	5	5	1.02			
12	1	41	41	3.5	5.-7	1.00			
13	1	45	44	4.5	6	1.02			
14	1	44	40	4.5	5	1.10			
15	1	45.4	41	3	5	1.11			
16	1	49	44	4	4.5	1.11			
**17**	**2**	**46**	**41**	**2.5–3**	**4**	**1.12**	**paratype 1**	**Szel 21b-17-P1**	**Pl.1.B**
18	2	50	47	3	8	1.06			
19	2	48	42	3.5	4	1.14			Pl.2.D
20	2	50	45	3.-4	3	1.11			
21	2	45	42	2.5	6	1.07			
22	2	42	40	3	5	1.05			
23	2	47.5	45	3	5	1.06			
24	2	48	43	3.5	8	1.12			
25	2	40	38	2	3.5	1.05			
26	2	42	40	3	6	1.05			
**27**	**2**	**39**	**36**	**2.5**	**3**	**1.08**	**HOLOTYPE**	**Szel 21b-27-H**	**Pl.1.A**
**28**	**2**	**52**	**52**	**5**	**3**	**1.00**	**paratype 4**	**Szel 21b-28-P4**	**Pl.1.E**
29	2	47	45	3	4	1.04			
30	2	43	41	3	3	1.05			
31	2	59	56	5	6	1.05			
32	2	55	50	5	7.-8	1.10			Pl.2.A
33	2	43	38	4	4.5	1.13			
34	2	47	43	6	8	1.09			
**35**	**3**	**50**	**48**	**3**	**2.5**	**1.04**	**paratype 2**	**Szel 21b-35-P2**	**Pl.1.C**
36	3	55	46	3	4.5	1.20			
37	3	40	37	2.5	3	1.08			
38	3	40	38	2	4	1.05			
39	3	39	36	2.5	5	1.08			
40	3	50	45	3	7	1.11			
41	3	42	38	4	4	1.11			
42	3	41	37	2	5	1.11			
43	3	40	40	2.5	6	1.00			
44	3	41	38	3	5	1.08			
45	3	40	39	2.5	4	1.03			
46	3	47	43	3	3.5	1.09			
47	3	51	49	4	5	1.04			
48	3	40	40	3	4.5	1.00			
49	3	51	49	2.5	5	1.04			
50	3	52	47	5	7	1.11			
51	4	52	41	3	8	1.27			
52	4	51	40	7	7	1.28			
53	4	50	45	4.5	6	1.11			
54	4	46	42	2.5	4	1.10			
55	4	44.5	44	4	5	1.01			Pl.3.B
56	4	50	43	3	6	1.16			
57	4	42	42	3	5	1.00			
58	4	50	43	2.5	4	1.16			
59	4	46	41	4	4.5	1.12			
60	4	40.5	40	5	6	1.01			
61	4	51	42	3	5	1.21			
62	4	41	37	2.5	5	1.11			
63	4	49	45.5	3	5	1.08			
64	4	43	42	4	4	1.02			
65	4	48	47	4	5.5	1.02			
66	4	49	46	4	6.5	1.07			
67	5	48	41	4	7	1.17			
68	5	50	45.5	5	8	1.10			
69	5	49	46	2.5	5	1.07			
70	5	52	48	5	6	1.08			
71	5	49	45	45	7	1.09			
72	5	50	50	5	6	1.00			
73	5	58	42	4	4	1.38			
74	5	49	45	2.5	6	1.09			
75	5	47	42	3	4	1.12			
76	5	52	52	5	5.5	1.00			
77	5	43	42	4	5	1.02			
78	5	41.5	41	3	4.5	1.01			
79	5	43	41	3	6	1.05			
80	5	48	48	2.5	5	1.00			
81	5	49	42	3	5	1.17			
**82**	**3**	**58**	**53**	**4**	**6**	**1.09**	**paratype 3**	**Szel 21a-82-P3**	**Pl.1.D**

L—length; W—width; TO—thickness of outer layer, TI—thickness of inner layer, Ec—elongation coefficient (Ec = L/W).

**Table 2:** Comparison of dimensions of the *Cadosinopsis rehakovii* sp. nov. and *Cadosinopsis nowaki* Borza, 1984, and *Cadosinopsis andrusovi* Scheibner, 1967.

Species	No.	L [μm]	W [μm]	TO [μm]	TI [μm]	Ec	Data source
*Cadosinopsis nowaki*	1	50	50	4	10	1.00	Borza, 1984, Table 1
	2	53	51	4.5	6	1.04	Borza, 1984, Table 1
	3	66	63	4	11	1.05	Borza, 1984, Table 1
	4	66	60	4	9	1.10	Borza, 1984, Table 1
	5	70	70	4	16	1.00	Borza, 1984, Table 1
	6	73	70	5	11	1.04	Borza, 1984, Table 1
*Cadosinopsis andrusovi*	7	108	80	7	12	1.35	Scheibner, 1967, Plate 1
	8	120	80	6	11	1.50	Scheibner, 1967, Plate 1
	9	100	70	6	13.5	1.43	Scheibner, 1967, Plate 1
	10	98	60	5	12.5	1.63	Scheibner, 1967, Plate 1
	11	68	68	5	10.5	1.00	Scheibner, 1967, Plate 1
	12	96	72	9	6	1.33	Scheibner, 1967, Plate 1
*Cadosinopsis rehakovii*	13	52	45	4	5	1.16	this paper, Szel 21b
sp. nov.	14	45	42	5	5	1.07	this paper, Szel 21b
	15	50	46	3	10	1.09	this paper, Szel 21b
	16	34	30	2.5	3	1.13	this paper, Szel 21b
	17	50	44	4	5	1.14	this paper, Szel 21b
	18	59	56	5	6	1.05	this paper, Szel 21b
	19	52	48	4	6	1.08	this paper, Szel 21b

L—length; W—width; TO—thickness of outer layer, TI- thickness of inner layer; Ec—elongation coefficient (Ec = L/W).

### Nomenclature

The electronic version of this article in Portable Document Format (PDF) in a work with an ISSN or ISBN will represent a published work according to the International Code of Nomenclature for algae, fungi, and plants, and hence the new names contained in the electronic publication of a PLOS ONE article are effectively published under that Code from the electronic edition alone, so there is no longer any need to provide printed copies. The online version of this work is archived and available from the following digital repositories: PubMed Central, LOCKSS.

## Results and discussion

### Calcareous dinocyst assemblage

Calcareous dinocysts were present in the studied section in the upper part of the Upszar Limestone Member and lower part of the Pieniny Limestone Formation. The first cysts appear 3.7 m below the clear boundary between the Upszar Limestone Member and the Pieniny Limestone Formation, in correspondence of the first intercalations of green limestone within red and variegated deposits. Generally, cysts are present throughout these variegated and green to grayish limestone. The assemblage of dinoflagellate cysts is abundant and diversified as it consists of a total of 17 species from eight genera. Precise age data and biostratigraphic analysis will be the subject of a separate publication. The assemblage contains common and diversified *Colomisphaera*, with seven species present along the entire series, especially *C*. *carpathica* (Borza), and *C*. *radiata* (Vogler). Other common species include *Carpistomiosphaera tithonica* Nowak, *Committosphaera pulla* (Borza), and *Crustocadosina semiradiata semiradiata* (Wanner).

This calcareous dinocyst assemblage indicates an early Tithonian age of these deposits. Precise age data and biostratigraphic analysis will be the subject of a separate publication. The upper part of the Upszar Limestone Member, with predominately green intercalations has been classified to the Tithonica Zone of Calcareous Dinoflagellate Zonation. The lowermost part of the Pieniny Limestone Formation outcropped in the studied section represents Malmica and Semiradiata zones of Calcareous Dinoflagellate Zonation.

### Systematic paleontology

Domain **Eukaryota** Chatton, 1925

Kingdom **Chromista** Cavalier-Smith, (1981)

Subkingdom **Harosa** Cavalier-Smith, 2010

Infrakingdom **Alveolata** Cavalier-Smith, 1991

Phylum **Miozoa** Cavalier-Smith, 1987

Subphylum **Myzozoa** Cavalier-Smith and Chao, 2004

Infraphylum **Dinozoa** Cavalier-Smith, 1981

Superclass **Dinoflagellata** (Bütschli, 1885) Fensome et al., 1993

Class **Dinophyceae** Pascher, 1914

Subclass **Peridiniphycidae** Fensome et al., 1993

Order **Peridiniales**, Haeckel, 1894

Family **Thoracosphaeracea** Schiller, 1930

Genus ***Cadosinopsis*** Scheibner, 1967

***Cadosinopsis rehakovii* sp. nov.** Ciurej at Bak, sp. nov.

(Figs 3–5A, Figs 2, 6–9)

**Fig 3:**
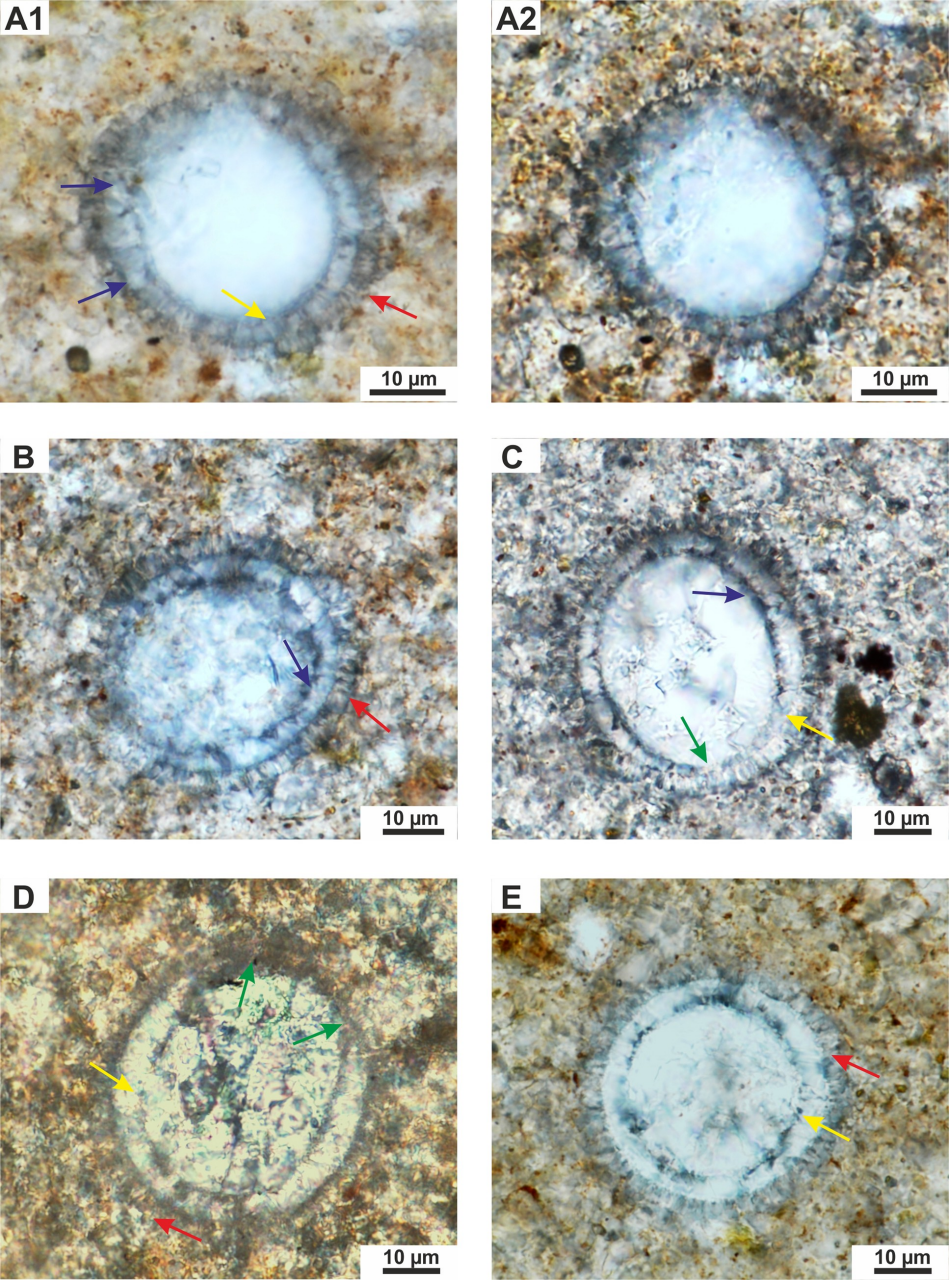
**A-E—***Cadosinopsis rehakovii* sp. nov. **A1,A2** –holotype—repository number: Szel 21b-27-H—The same view showing the longitudinal section of a cyst and morphology of two layers: outer (red arrow) composed of the short fibrous calcite crystals and inner (yellow arrow) composed of the coarse, thick plate-shaped calcite crystals. Note the local diagenetic (?) calcite overgrowth in the inner layer (blue arrow). TS. Szel 21b. A1- 1N, A2-XN, SCA, LDC. **B**–paratype 1—repository number: Szel 21b-17-P1. Longitudinal section with well visible inner layer with coarse calcite crystals, and outer layer with fibrous calcite (red arrow), locally less visible. Note the dark thin “film” on proximal side of the inner layer (blue arrow). TS. Szel 21b.1N, SCA, LDC. **C**–paratype 2—repository number: Szel 21b-35-P2. Longitudinal section with well visible inner layer (yellow arrow) with aperture (green arrows). Note the dark thin “film” on proximal side of the inner layer (blue arrow). TS. Szel 21b.1N, SCA, LDC. **D**–paratype 3—repository number: Szel 21a-82-P3. Longitudinal section with well visible inner layer (yellow arrow) with wide aperture (pointed by green arrows). The outer layer is very difficult to see (red arrow). TS. Szel 21a.1N, OA, LDC. **E**—paratype 4—repository number: Szel 21b-28-P4. Transversal section showing the circular shape of a cyst with well-preserved inner (yellow arrow) and outer layer (red arrow). Note the equal thickness of the inner layer. TS. Szel 21b. 1N, SCA.

**Fig 4:**
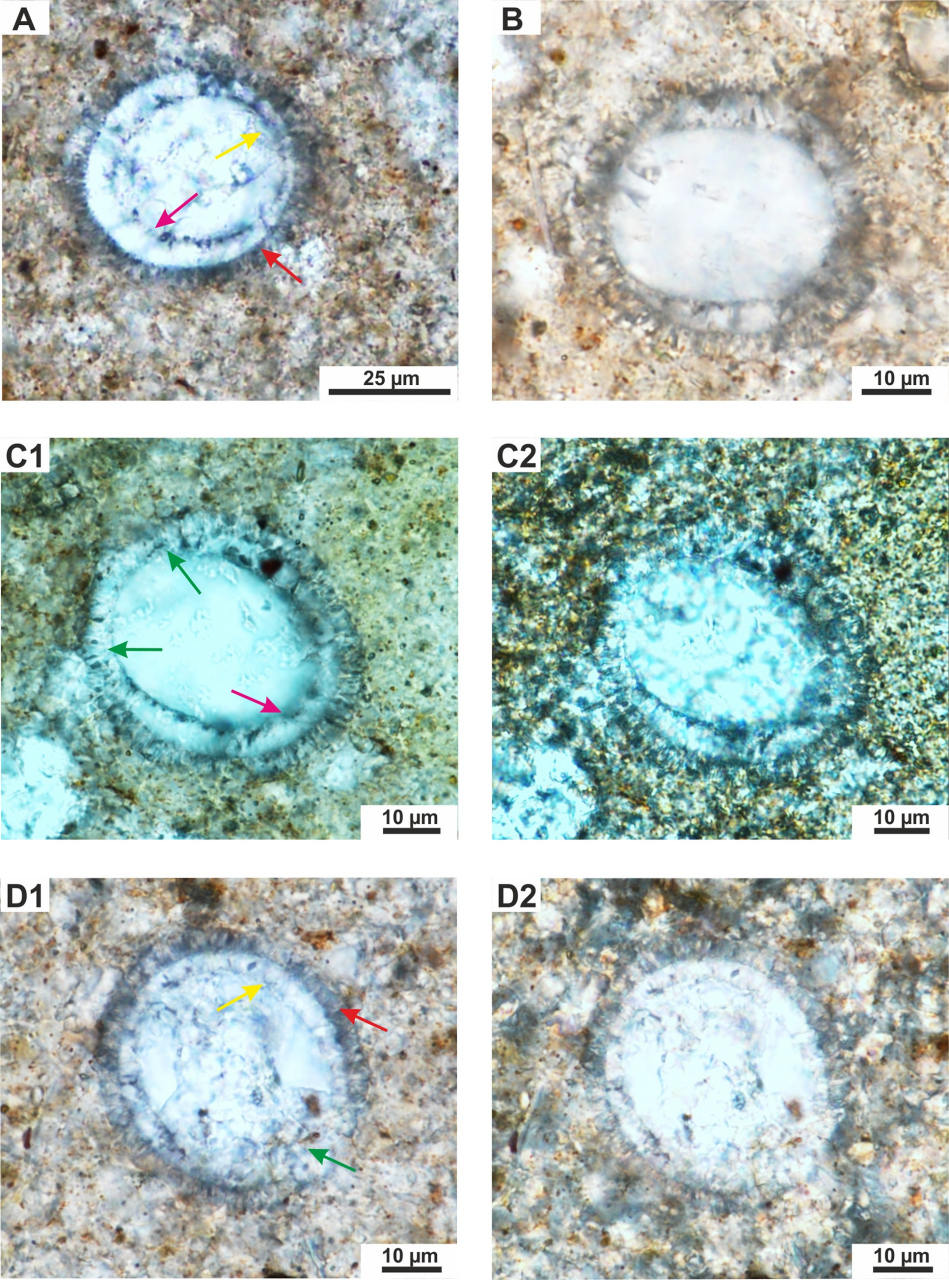
A-D–*Cadosinopsis rehakovii* sp. nov. **A–**longitudinal section with well visible outer layer (red arrow) and inner layer with not equal thickness—the thinnest (yellow arrow) is in the adjacent area of the aperture. Note the even and smooth proximal margin of the inner layer (pink arrow). Szel 21b-32 in Table 1. TS. Szel 21b.1N, OA, LDC. **B–**longitudinal sections with inner and outer layers, hardly visible in host rock. Szel 21b-41 in Table 1. TS. Szel 21b.1N OA, LDC. **C1-C2 –**the same view in 1N and XN, respectively, showing longitudinal section of cyst with the thick inner layer, locally slightly ragged in proximal margin (pink arrow), The wide aperture is observed (green arrows). Szel 21b-1 in Table 1. TS. Szel 21b. C1- 1N, OA, LDC and C2 –XN, SCA, LDC. **D1-D2** –longitudinal section with well preserved outer (red arrow) and inner layer (yellow arrow), with slightly diagenetically obliterated aperture side (green arrow). Szel 21b-19 in Table 1. TS. Szel 21b.D1-1N, D2-XN, OA, LDC.

**Fig 5:**
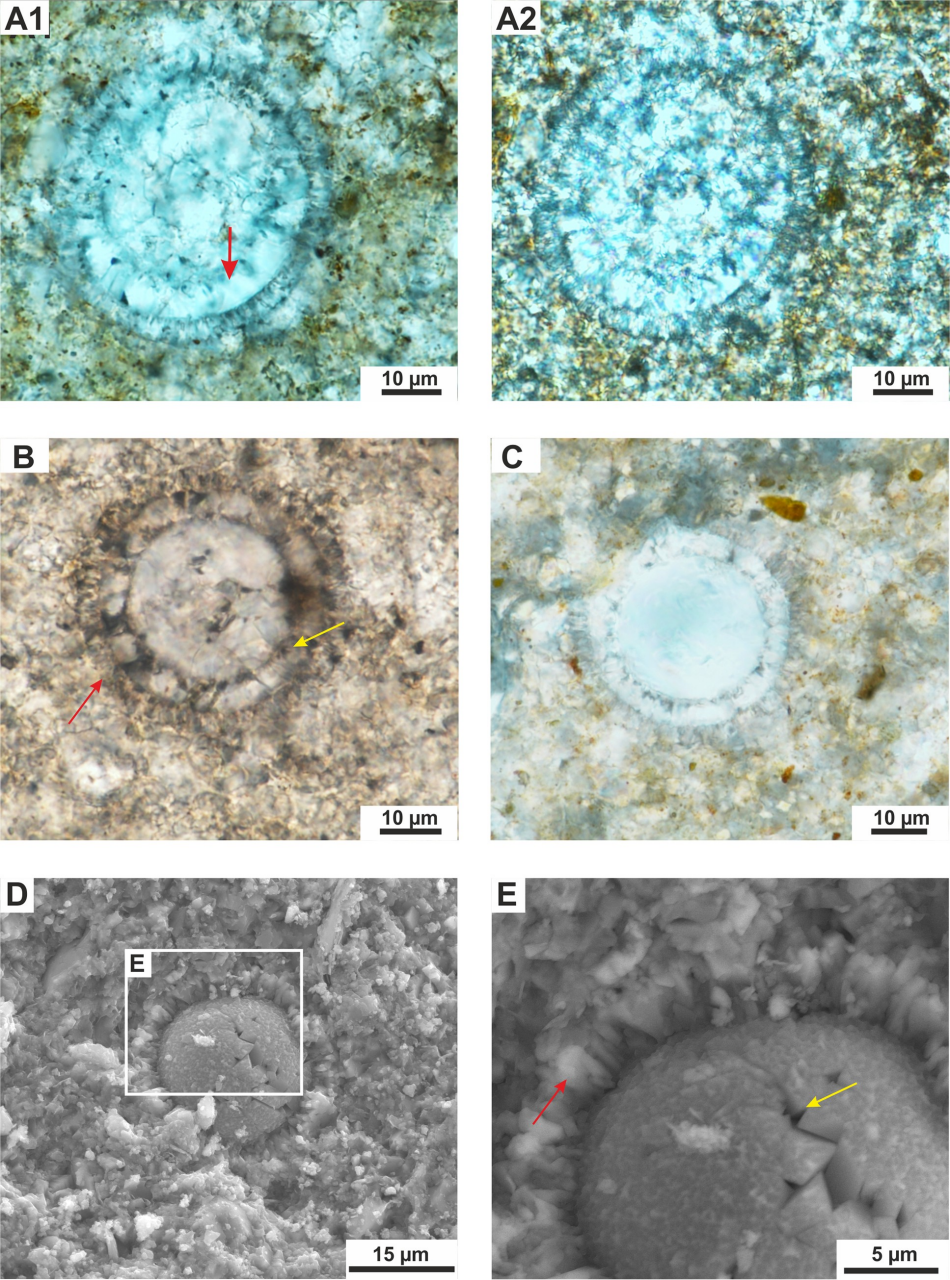
**A-E—***Cadosinopsis rehakovii* sp. nov. **A1-A2 –**longitudinal section—the same view of advanced diagenetic on the inner layer with calcite overgrowth, note uneven proximal side of inner wall (arrow), hardly distinguishable from inside filling. Szel 21b-10 in Table 1. TS. Szel 21b. A1- 1N, SCA and A2 –XN, LDC. **B–**transversal section of a cyst with well visible, thick inner layer and hardly recognizable from the surrounding material outer layer. Szel 21b-55 in Table 1.TS. Szel 21b.1N. OA, LDC. **C**—transversal section of a cyst showing the thick inner and thin outer layer. Szel 21a. TS. Szel 21a. XN. OA, LDC. **D**—SEM photomicrograph of transversal section of cyst showing the structure of outer and inner layers, enlarged on Fig E. Rock chips. Szel 21b. **E**—SEM photomicrograph of enlarged image from Fig D showing the outer layer with radially oriented, short, fibrous calcite crystals (red arrow) and inner layer with coarse of a thick, plate-shaped calcite crystals (yellow arrow).

**Fig 6:**
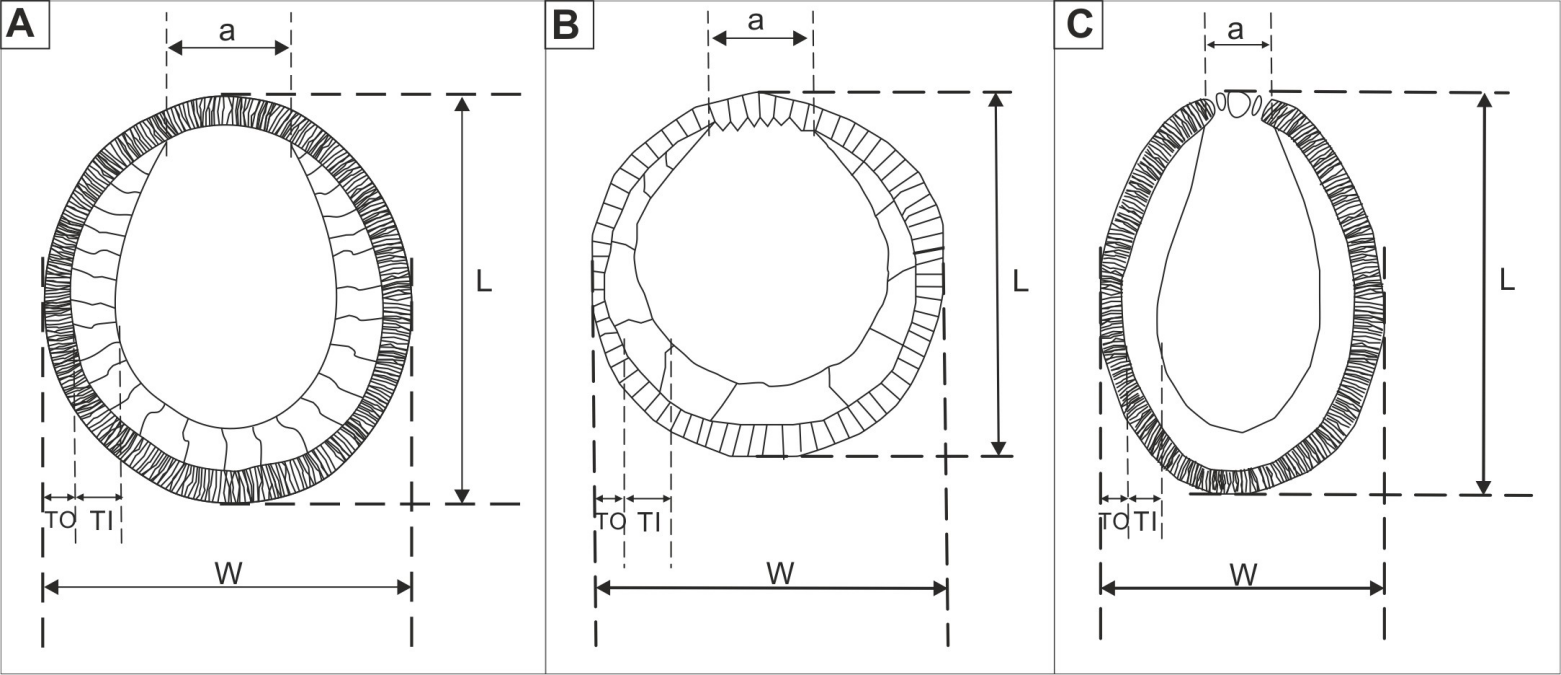
Schematized outline of test with two layers (outer and inner) in longitudinal section of (A) *Cadosinopsis rehakovii* sp. nov. (based on holotype and paratypes cross-sections), (B) *Cadosinopsis nowaki* Borza, 1984, (based on Borza, 1984), (C) *Cadosinopsis andrusovi* Scheibner, 1967 (based on Scheibner, 1967). Not for scale. L–length, W–width, TO–greatest thickness of outer layer, TI–thickness of inner layer, a–aperture.

**Fig 7:**
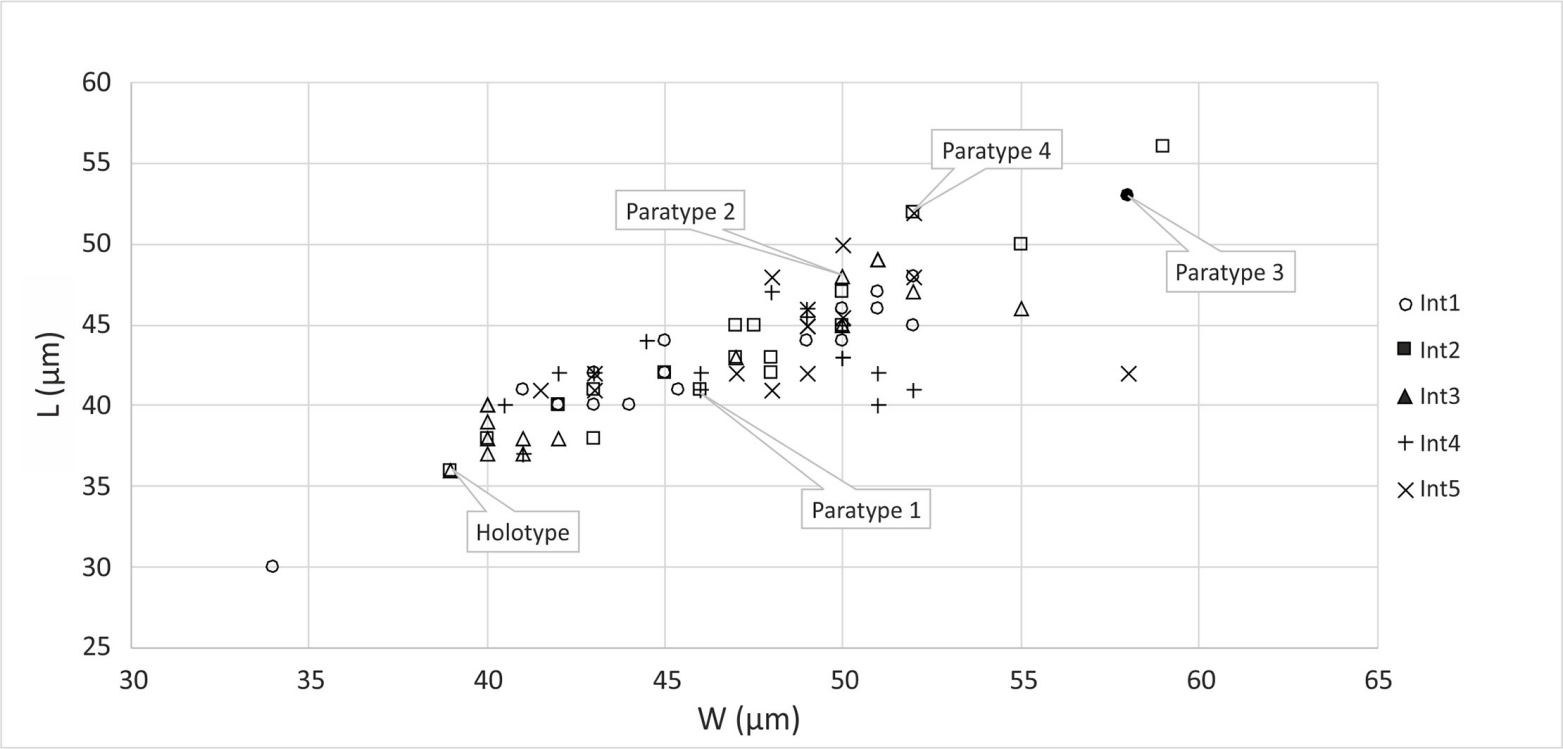
Diagram representing the relation between the length (L) and width (W) of *Cadosinopsis rehakovii* sp. nov., based on data from Table 1. **Int 1—Int 5 –intervals separated on the studied thin section no, Szel 21b.** Thin sections no: Szel 21a and Szel 21b.

**Fig 8:**
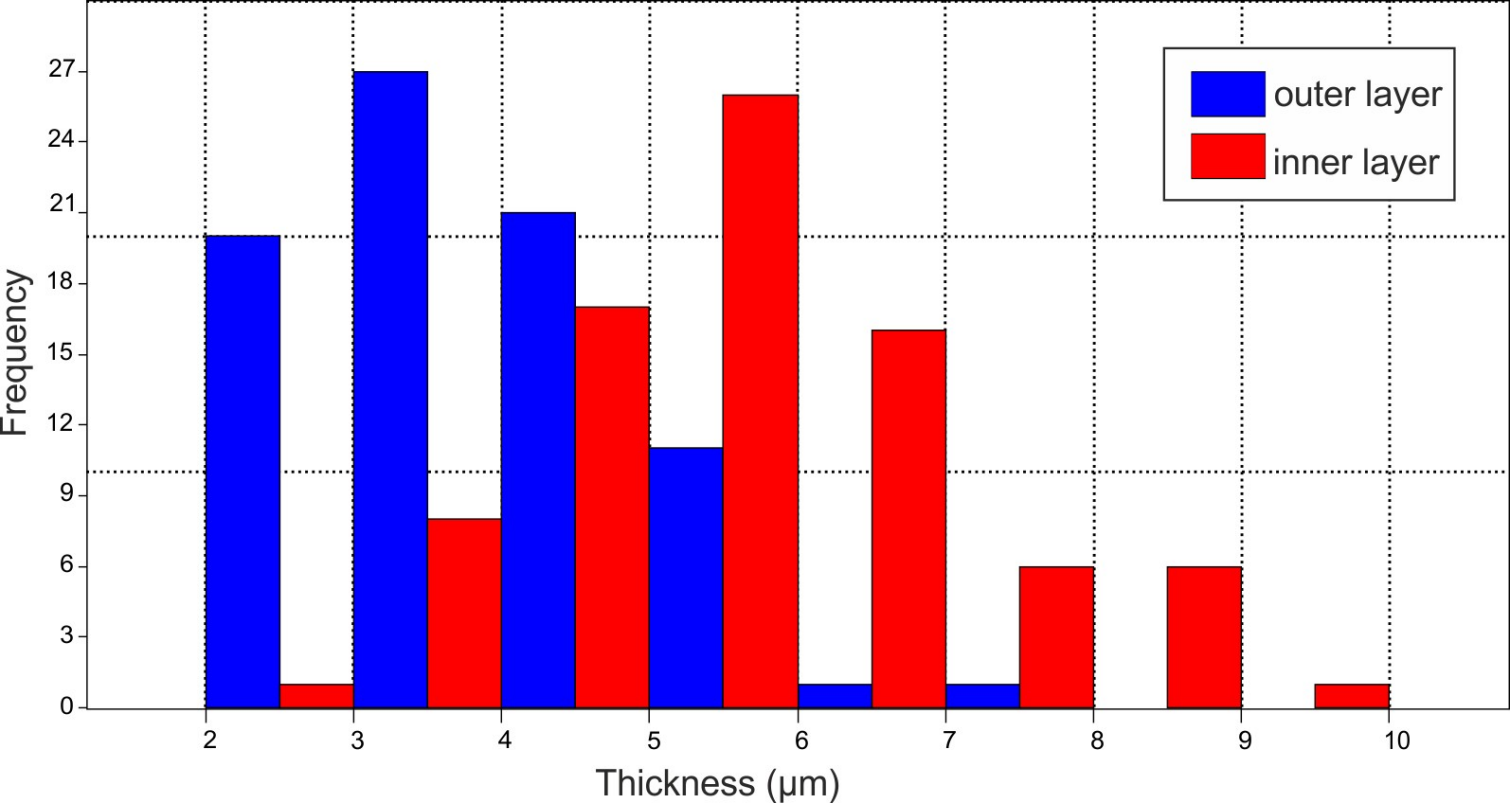
Thickness frequency of individual layers in the wall of the species cysts *Cadosinopsis rehakovii* sp. nov.

**Fig 9:**
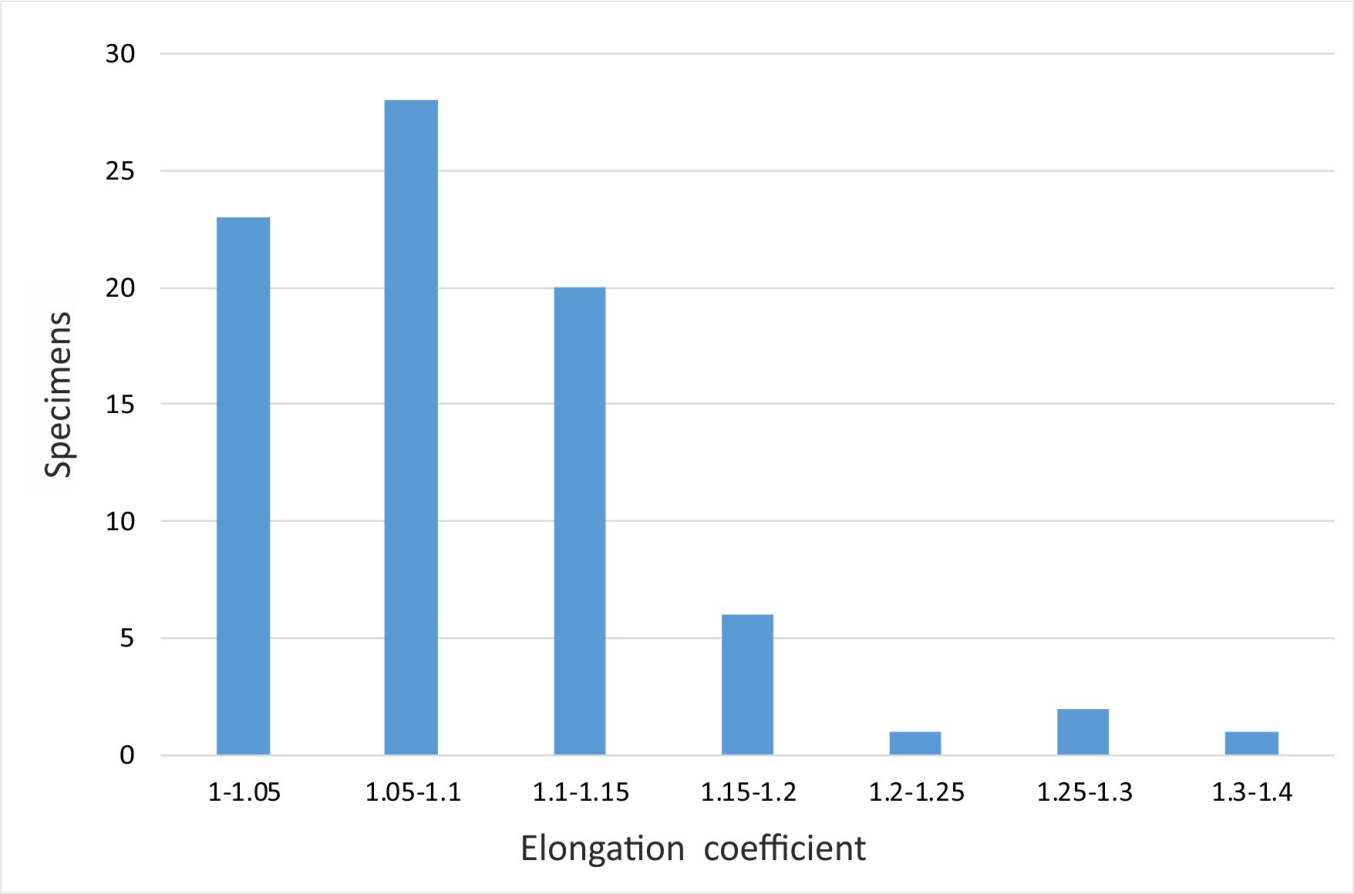
Diagram representing the elongation coefficient (Ec = L/W) of *Cadosinopsis rehakovii* sp. nov.

#### Derivation of name

In honor of Prof. Daniela Reháková for her significant contributions to calcareous dinocysts worldwide study.

#### Holotype

Species figured in Fig 3A, repository number Szel 21b-27-H, thin section, No. Szel 21b, collection reference: EMRC 7/11A

#### Paratype 1

Species figured in Fig 3B, repository number Szel 21b-17-P1, thin section, No. Szel 21b, collection reference: EMRC 7/11A **Paratype 2:** species figured in Fig 3C, repository number Szel 21b-35-P2, thin section, No. Szel 21b, collection reference: EMRC 7/11A **Paratype 3**: species figured in Fig 3D, repository number Szel 21a-82-P3, thin section, No. Szel 21, collection reference: EMRC 7/11A

#### Paratype 4

Species figured in Fig 3E, repository number Szel 21b-28-P4, thin section, No. Szel 21b, collection reference: EMRC 7/11A

#### Locality and horizon

Szeligowy Creek in the Nowy Targ Depression in southern Poland; the Branisko Succession in the Pieniny Klippen Belt; greenish to variegate limestone in the upper part of the Upszar Limestone Member of the Czorsztyn Limestone Formation; first appearance 3,7 m below the lower boundary of the Pieniny Limestone Formation.

#### Material

Approximately 231 specimens in various cross-section from thin sections.

#### Diagnostic

Microfossil with calcareous test spherical to oval, with wall composed of two layers: inner layer built of coarse calcite crystals and outer layer, thinner, built of the short fibrous calcite crystals. The chamber is located in central part of the cyst. The aperture is wide and seen only in the inner layer (Fig 6A and Figs 3–5).

#### Description (concerns the features of specimens in thin sections of the rock sample)

Cyst is spherical to oval in cross section. The chamber is located centrally. The cyst is surrounded by a calcareous wall that consists of two layers: inner and outer layers (Fig 6A)

The inner layer is compose of thick, plate-shaped calcite crystals, of, about 4 to 6 μm wide (sparite) arranged radially to cyst surface (Figs 3–5). In transmitted light this layer has a milky/white color. The thickness of the inner layer is not equal in the longitudinal sections of the cyst. The wall is thickest in the aboral part and gradually thinning towards the aperture. In transversal sections of the cyst the thickness of the inner layer is equal (Figs 3E, 5B–5E). The aperture is observed in this layer. It is large and its width is up to 1/2 of test diameter (however the width of aperture is changing depending of the cyst section) (Fig 3A, 3B and 3D). Proximal side (proximal margin) of the inner layer is even, smooth rarely to slightly ragged. The dark thin “film” is often seen on this margin (Fig 3B and 3C). However, sometimes, the proximal side can be highly ragged, and uneven, related to advanced recrystallization of calcite crystals (Fig 5A). The distal side (distal margin) of the inner layer is smooth and even. The boundary with outer layer is well visible.

Outer layer is built of short, thin, fibrous, calcite crystals radially arranged to the cyst surface (Figs 3–5). In transmitted light this layer is vitreous (transparent). This thickness is equal, regardless of the cross section of the cyst (Fig 3A and 3B, and Fig 5B–5E). The proximal and distal side of the outer layer is rather even, rarely uneven/jagged. No dark axial cross is observed under the crossed polarizers (Fig 3A; Fig 4C and 4D and Fig 5A and 5C). No aperture is observed in the outer layer.

#### Dimensions

**Fig 10:**
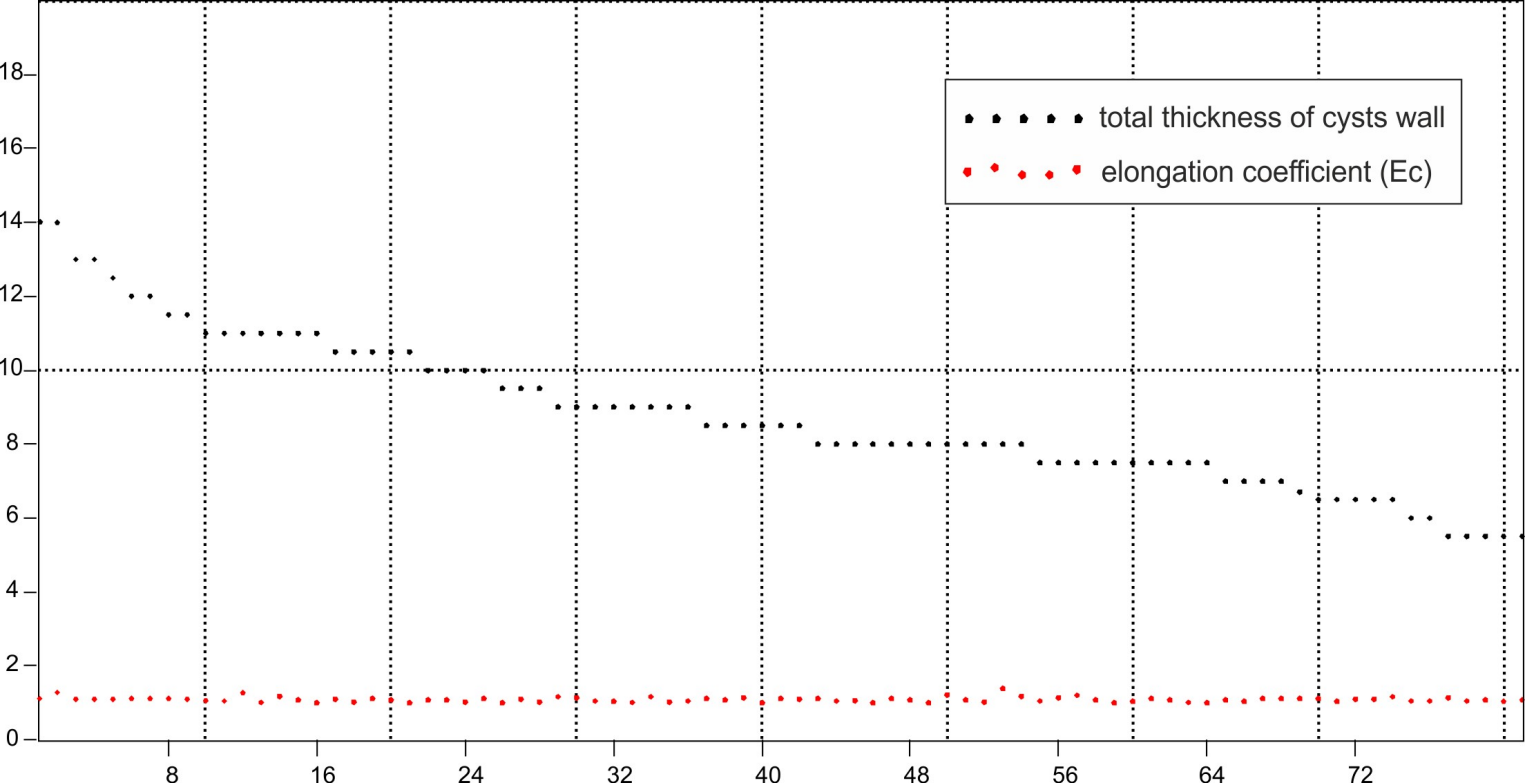
Diagram representing the relation between the total thickness of cysts wall and elongation coefficient (Ec = L/W) of *Cadosinopsis rehakovii* sp. nov.

**Fig 11:**
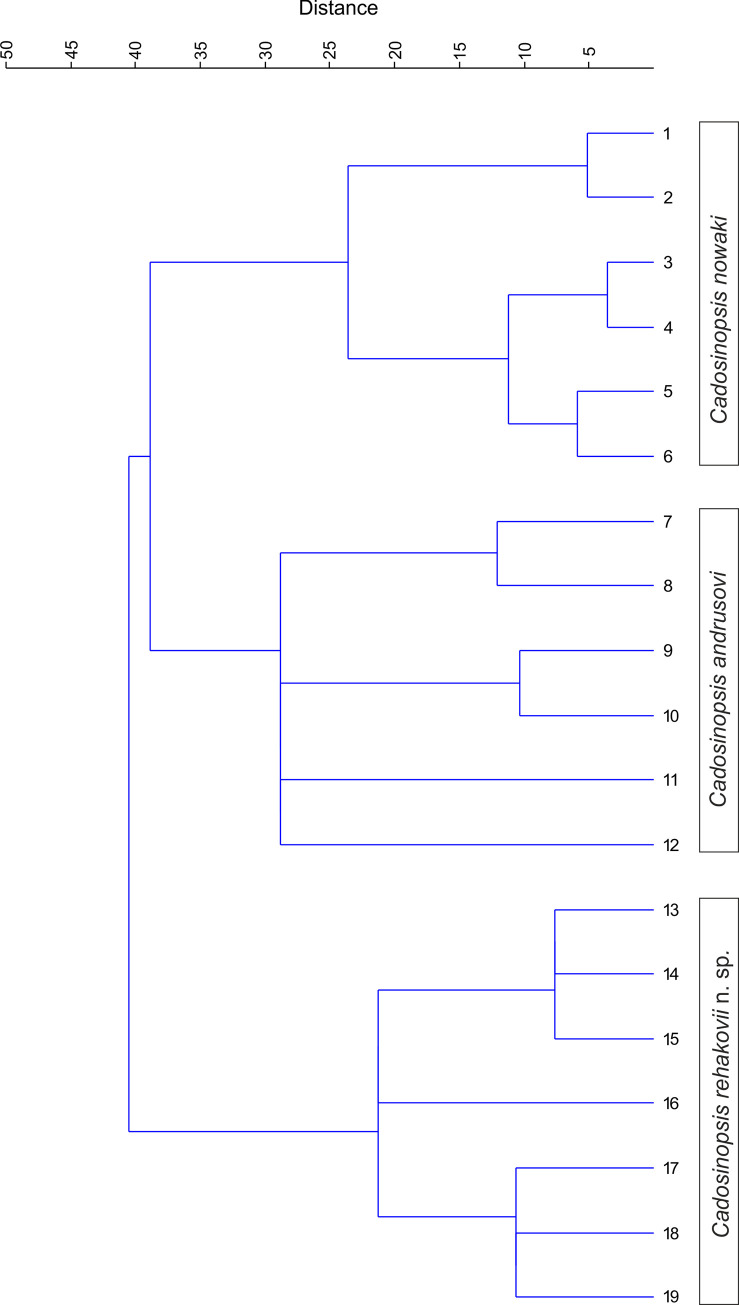
Cluster diagram for two already recognized species of Cadosinopsis genus (*C*. *nowaki* and *C*. *andrusovi*) and newly defined *C*. *rehakovii*, based on Ward’s method [58].

The parameters of studied specimens are presented in Figs 8–11. and Table 1. Length of cyst varies from 34 to 59 μm, width varies from 30 to 50 μm (Fig 7).

The outer layer is thinner than the inner layer. The thickness of the inner layers range from 2.5 to 10 μm. The thickness of the outer layer range from 2 to 7 μm. Thickness frequency of individual layers in the wall of the species is shown on Fig 8.

The elongation coefficient (Ec) was calculated as the ratio of the length (L) to the width of the cyst (W). This parameter varies from 1.05 to 1.40 for the 82 specimens measured (Fig 9). The most abundant values are within the range of 1.05 to 1.15. Diagram representing relation of the total thickness of cyst wall and elongation coefficient is shown on Fig 10.

#### Stratigraphic range

Lower Tithonian in the Pieniny Klippen Belt, present in the Tithonica Calcareous Dinoflagellate Zone [33].

#### Remarks

The new species shows some morphological similarities to the *Cadosinopsis nowaki* Borza 1984. The shape of cyst and position and development of aperture are similar (Fig 6B). However *C*. *nowaki* has a bigger cyst, the length ranges from 50 to 76 μm and width ranges 43 to 67 μm. The inner layer is also thicker (up to 17 um). The chamber of *C*. *nowaki* is slightly offset from the center. The inner layer is also built of the coarse-sparite calcite crystals but it is vitreous in transmitted light and the outer layer is also built of the fibrous but it is white in transmitted light [36, 50]. The stratigraphic position is also different. The stratigraphic position is also different—upper Tithonian [51] and Santonian-Campanian [50].

*Cadosinopsis andrusovi* Scheibner 1967 is also morphologically similar, although it is much bigger as its cysts length ranges from 68 to 108 μm and width ranges from 60 to 80 μm. The cyst is more oval and has a less spherical chamber. The outer layer contains few pores on the aperture side (Fig 6C). The stratigraphic position is different as it occurs in deposits of Santonian age [49].

Similarities in morphology are also observed in *Crustocadosina semiradiata olzae* Nowak, 1996. However this species is often bigger, has the very thick, micritic inner layer which is dark in transmitted light. The outer layer of this species consists of short calcite crystals and is white in transmitted light. The chamber is more oval and more eccentrically located in the cyst. The stratigraphic position of this species is also different from the newly assigned *Cadosinopsis rehakovii* sp. nov.-Tithonian—Late Valanginian [33].

*Cadosinopsis rehakovii* sp. nov when observed in transverse section can display morphological similarities with *Parasotmiosphaerea malmica* Borza, 1964. However, *P*. *malmica* species differs in the character of layers: the inner is composed of calcite crystals with c-axis arranged diagonally in the cyst surface, and is slightly yellow and brown in transmitted light. The outer layer has radially arranged calcite crystals, milky in transmitted light and showing an axial cross under the crossed polars. The stratigraphic position is also different, from middle part of Tithonian [33, 36].

The *Carpistomiosphaera borzai* (Nagy 1966), show morphological similarities to *C*. *rehakovi* sp. nov. especially when the latter one is less preserved and observed in transverse section. Both occur in the same stratigraphic position. However, both layers of *C*. *borzai* have a similar thickness, and are built of short calcite crystals, white in transmitted light [61].

Some morphological similarities are also observed in *Parasotmiosphaerea tithonica* Nowak 1968, which occurs in the same stratigraphic position (Tithonian) (e.g. [29, 44]). However this specimen has an outer layer thicker than the inner layer, which is the opposite of *C*. *rehakovii* sp. nov. The inner layer is slightly dark, and composed of short calcite crystals, and outer layer is built of long calcite crystals.

### Attribution of a new species to the genus *Cadosinopsis*

The newly recognized calcareous dinocysts classified herein as a new species have been included in the genus *Cadosinopsis* on the basis of the structure of the cyst wall made of two layers and the specific arrangement of calcite crystals in these layers. The inner layer is built of the sparite calcite crystals and the outer layer is built of the fibrous and radially arranged calcite crystals. The genus *Cadosinopsis* differs from *Carpistomiosphaera* by cross-like inter-fingering calcite crystals [62]. Calcite crystals in this genus are tiny and their axes are oblique or tangent to the cyst surface which is not observed in specimens of the new species. Species of the genus *Colomisphaera* containing one layer, composed of calcite crystals obliquely arranged in cyst surface. It can be short and thick (*C*. *lapidosa*) or strongly elongated (*C*. *carpathica*) [62]. This layer is vitreous in transmitted light, but it gives a non-translucent image, which is related to the relief effect resulting from the different optical orientation of the c axis of the calcite crystals with respect to the cyst surface.

The new species have been compared with other species of *Cadosinopsis* as *C*. *nowaki* and *C*. *andrusovi*. The features on the basis of which the grouping was made are presented in Table 2.

Cluster analysis was employed to find hierarchical groupings in the dataset containing specimens of two already existing species of *Cadosinopsis* genus and the newly defined *Cadosinopsis rehakovii*. The dendrograms derived from Ward’s method (with Euclidean distance) and the unweighted pair–group average (computed separately with Chord distance and Morisita’s index) were compared. As the groupings were effectively the same after using these methods, only one dendrogram, constructed using Ward’s method, is presented on Fig 11.

The hierarchical clustering routine (R-mode) produced a cluster diagram showing three separate groups compatible with the distinguished species of genus *Cadosinopsis* ‘Group one’ assembled specimens of *C*. *nowaki* previously distinguished by Borza in his study; ‘Group two’ combined specimens of *C*. *andrusovi* derived from Scheibner’s study. Group three agglomerated Carpathian specimens, which have been nominated as new species–*C*. *rehakovii*. Cluster analysis also shows a degree of similarity between distinguished groups of specimens. The specimens of *C*. *nowaki* (‘Group one’) showed close similarity with *C*. *andrusovi*. Subsequently, *C*. *rehakovii* is combined further with the *C*. *nowaki–C*. *andrusovi* ‘supercluster’. It indicates that *C*. *nowaki* and *C*. *andrusovi* are morphologically much closer than the newly distinguished *C*. *rehakovii* from the Carpathians assemblage. Both species consist of separate branches of the dendrogram.

**Fig 12:**
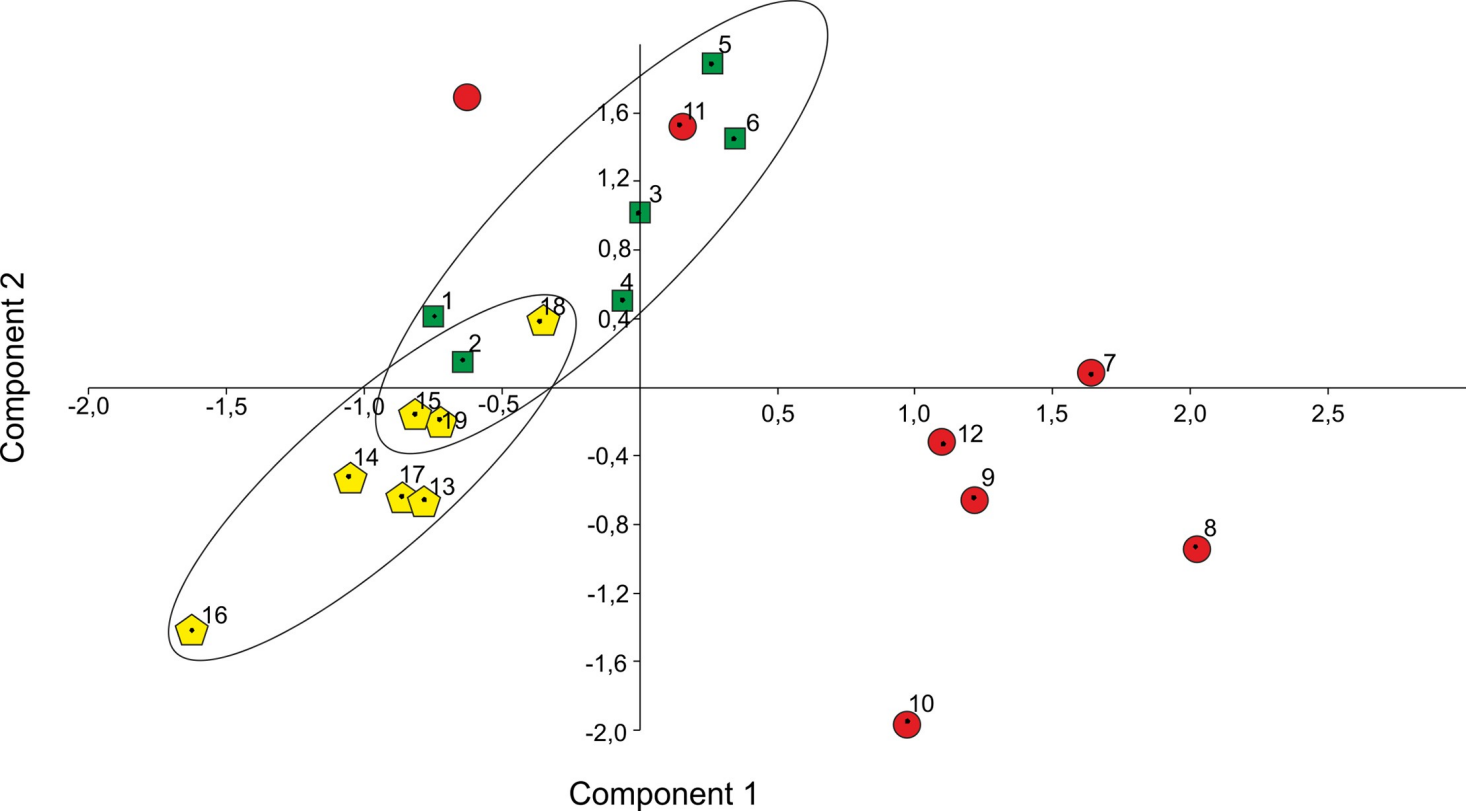
Principal Component Analysis (PCA). Plot of coordinates given by the two most important components. The percentages of variance accounted are given in the text. 1–6 –*Cadosinopsis nowaki*, 7–12—*C*. *andrusovi* and 13–19*—*newly defined *C*. *rehakovii*.

Principal component analysis (PCA) was carried out the whole dataset (Table 2), with all specimens included in one PCA analysis, identifying major axes of morphological variation. The PCA routine distinguished the eigenvalues and eigenvectors of the variance–covariance matrix. The eigenvalues gave a measure of the variance accounted for by the corresponding components. The percentage of variance accounted for by the first four most important components are: (1) 94.7; (2) 4.4; (3) 0.9; (4) 0.05. The PCA results score each species along a series of abstract mathematical axes of decreasing statistical importance. Component-1 and component-2, with highest variance, were plotted against one another to show grouping trends in the dataset (Fig 12).

**Fig 13:**
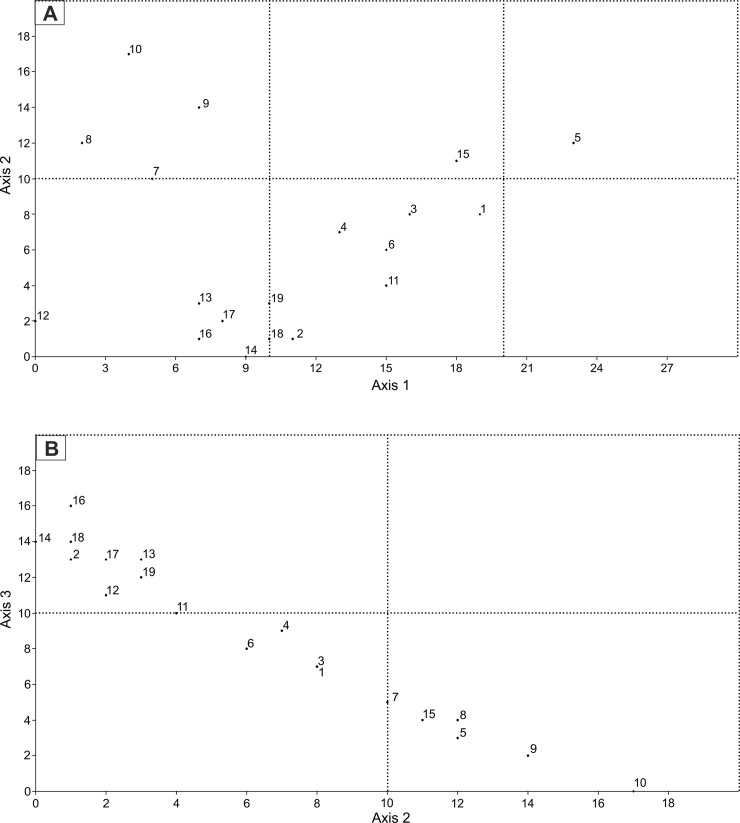
Scatter plot of three groups of specimens (previously distinguished in Table 2) in Canonical Variates Analysis (CVA). A—calculated on the basis of column 3–5 from Table 2, which illustrated measurements of thickness of inner and outer layer in relation to cyst shape coefficient, B–calculated on the basis of column 3–4 from Table 2 which shows that increasing cyst size implies thicker inner and outer layers. 1–6 –*Cadosinopsis nowaki*, 7–12—*C*. *andrusovi* and 13–19—newly defined *C*. *rehakovii*.

The first principal component is the most important, explaining 94.7% of the variation. All calculated distances increase almost equally fast. It indicates that the first axis captures more stable parameters which is general size. Loadings on the second component showed that, axially, these measurements are variable especially when the forms change the shape from oval to elongated (e.g. length of whole specimens in perpendicular direction to the first parameters which rapidly decrease with width values). The second component can therefore describe elongation of the cysts. The use of the first two components reduces the original multi-dimensional dataset to two dimensions. Constructed scatter plots (Fig 13) showed that three groups of specimens, previously distinguished in Table 2, occupy three almost different regions of morphospace. Separation between the groups on the PCA scatter plot has been corroborated additionally using CVA (Fig 13A) calculated on the basis of column 3–5 from Table 2, which illustrated measurements of thickness of inner and outer layer in relation to cyst shape coefficient (Ec). Deviations from this rule are probably the result of diagenetic changes in crystal size of the inner or outer layer. This may be the result of the crystals growing or dissolving, which is not clearly observable during micropaleontological analysis. One of the examples is *C*. *nowaki* illustrated by Borza [50] (specimens No. 2 in Table 2) which has very thin inner layer in relation to other specimens of *C*. *nowaki* (for example see Table 2). Another example may be newly defined *C*. *rehakovii* (No. 15) which possesses very thick inner layer in relation to another specimens. The CVA analysis additionally shows that increasing cyst size implies thicker inner and outer layers. Points from three designated groups are arranged linearly on the diagram (Fig 13B).

## Conclusions

The Lower Tithonian deposits within the Branisko succession of the Pieniny Klippen Belt (Western Carpathians) are rich in microfossil of *Cadosinopsis rehakovii* sp. nov. which yield common features with the genus *Cadosinopsis*. Microscopic examination by optical microscope and SEM provides diagnostic criteria of *Cadosinopsis rehakovii* sp. nov. that may be summarized as follows:

- calcareous test spherical to oval, with length from 34 to 59 μm and width from 30 to 50 μm,
- chamber located in the central part of the cyst,
- wall composed of two layers,
- outer layer, 2 to 7 μm thick built of short fibrous calcite crystals radially arranged to the cyst surface and vitreous (transparent), in transmitted light,
- inner layer, from 2.5 to 10 μm thick, composed of coarse, thick, plate-shaped calcite crystals, radially arranged and milky/white colour in transmitted light,
- the aperture is wide and seen only in the inner layer,
- no dark axial cross is observed under the crossed polars,

## References

[1] Williamson WC. On the organization of the fossil plants of the coalmeasures. Part X–including an examination of the supposed radiolarians of the Carboniferous rocks. Philos Trans R Soc London. 1980;171:493–539.

[2] Kaźmierczak J, Kremer B. Early post-mortem calcified Devonian acritarchs as a source of calcispheric structures. Facies. 2005;51:573–584.

[3] Versteegh GJM, Servais T, Streng M, Munnecke A, Vachard D. A discussion and proposal concerning the use of the term calcispheres. Palaeontology. 2009;52(2):343–348.

[4] Tangen K, Brand LE, Blackwelder PL, Guillard RRL. *Thoracosphaera heimii* (Lohmann) Kamptner is a dinophyte: Observations on its morphology and life cycle. Mar Micropaleontol. 1982;7(3):193–212.

[5] Binder BJ, Anderson DM. Physiological and environmental control of germination of *Scrippsiella trochoidea* (Dinophyceae) resting cysts. J Phycol. 1987;23(1):99–107.

[6] Montresor M, Montesarchio E, Marino D, Zingone A. Calcareous dinoflagellate cysts in marine sediments of the Gulf of Naples (Meditenanean Sea). Rev Palaeobot Palynol. 1994;84:45–56.

[7] Fensome RA, Williams GL. The Lentin and Williams index of fossil dinoflagellates. College Park: American Association of Stratigraphic Palynologists.; 2004. 10.1016/j.bmcl.2004.02.054

[8] Keupp H. Fossil calcareous dinoflagellate cysts. In: Calcareous Algae and Stromatolites. Berlin: Springer; 1991. p. 267–286.

[9] Lewis J. Cyst-theca relationships in Scrippsiella (Dinophyceae) and related orthoperidinioid genera. Bot 3. 1991;34:91–106.

[10] Montresor M. *Scrippsiella ramonii* sp. nov. (Peridiniales, Dinophyceae), a marine dinoflagellate producing a calcareous resting cyst. Phycologia. 1995;34:87–91.

[11] Vink A. Calcareous dinoflagellate cysts in South and equatorial Atlantic surface sediments: Diversity, distribution, ecology and potential for palaeoenvironmental reconstruction. Mar Micropaleontol. 2004;50:43–88.

[12] Zonneveld KAF, Meier KJS, Esper O, Siggelkow D, Wendler I, Willems H. The (palaeo) environmental significance of modern calcareous dinoflagellate cysts: a review. Paläontologische Zeitschrift. 2005;79(1):61–77.

[13] Janofske D. Kalkiges Nannoplankton, insbesondere kalkige Dinoflagellaten-Zysten der alpinen Ober-Trias: Taxonomie, Biostratigraphie und Bedeutung für die Phylogenie der Peridiniales. Berliner geowissenschaftliche Abhandlungen. 1992;E 4:1–53.

[14] Dias-Brito D. Global stratigraphy, palaeobiogeography and palaeoecology of Albian-Maastrichtian pithonellid calcispheres: Impact on Tethys configuration. Cretac Res. 2000;21(2–3):315–349.

[15] Wendler J, Willems H. The distribution of pattern of calcareous dinoflagellate cysts at the K/T boundary (Fish Clay, Stevns Klint, Denmark)—Implications for our understanding of species selective extinction. Spec Pap Geol Soc Am. 2002;356:265–276.

[16] Wendler J, Wendler I, Willems H. *Orthopithonella collaris* sp. nov., a new calcareous dinoflagellate cyst from the K/T boundary (Fish Clay, Stevns Klint/Denmark). Rev Palaeobot Palynol. 2001;115(1–2):69–77. 10.1016/s0034-6667(01)00050-1 11425348

[17] Fensome RA. A classification of living and fossil dinoflagellates. Micropaleontol Spec Publ. 1993;7:1–245.

[18] Meier KJS, Young JR, Kirsch M, Feist-Burkhardt S. Evolution of different life-cycle strategies in oceanic calcareous dinoflagellates. Eur J Phycol. 2007;42(1):81–89.

[19] Elbrächter M, Gottschling M, Hildebrand-Habel T, Keupp H, Kohring R, Lewis J, et al. Establishing an agenda for calcareous dinoflagellate research (Thoracosphaeraceae, Dinophyceae) including a nomenclatural synopsis of generic names. Taxon. 2008;57(4):1289–1303.

[20] Keupp H, Mutterlose J. Organismenverteilung in den D-Beds von Speeton (Unterkreide, England) unter besonderer Berücksichtigung der kalkigen Dinoflagellaten-Zysten. Facies. 1984;10:153–178.

[21] Keupp H, Versteegh GJM. Ein neues Konzept für kalkige Dinoflagellaten-zysten der Subfamilie Orthopithonelloideae Keupp 1987. Berliner Geowissenschaftliche Abhandlungen. 1989;106(A):209–219.

[22] Young JR, Bergen JA, Bown PR, Burnett JA, Fiorentino A, Jordan RW, et al. Guidelines for coccolith and calcareous nannofossil terminology. Palaeontology. 1997;40(4):875–912.

[23] Fensome RA, Saldarriaga JF, Taylor MFJR. Dinoflagellate phylogeny revisited: Reconciling morphological and molecular based phylogenies. Grana. 1999;38(2–3):66–80.

[24] Janofske D, Karwath B. Oceanic calcareous dinoflagellates of the equatorial Atlantic Ocean: Cyst-theca relationship, taxonomy and aspects on ecology. In: Karwath B, editor. Ecological Studies on Living and Fossil Calcareous Dinoflagellates of the Equatorial and Tropical Atlantic Ocean. 152nd ed. Universität Bremen; 2000. p. 94–136.

[25] Streng M, Hildebrand-Habel T, Willems H. Proposed classification of archeopyle types in calcareous Dinoflagellate cysts. J Paleontol. 2004;78(3):456–483.

[26] Gottschling M, Keupp H, Plötner J, Knop R, Willems H, Kirsch M. Phylogeny of calcareous dinoflagellates as inferred from IST and ribosomal sequence data. Mol Phylogenet Evol. 2005;36:444–455. 10.1016/j.ympev.2005.03.036 15964218

[27] Kohring R, Gottschling M, Keupp H. Examples for character traits and palaeoecological significance of calcareous dinoflagellates. Paläontologische Zeitschrift. 2005;79(1):79–91.

[28] Nowak W. *Pithonella ovalis* (Kaufmann) dans les Carpates de Flysch occidentales (in Polish with French summary). Ann la Soc Geol Pologne. 1963;33:229–238.

[29] Nowak W. Stomiosphaerids of the Cieszyn Beds (Kimmeridgian–Hauterivian) in the Pol ish Cieszyn Silesia and their stratig raphic al value. Ann la Soc Geol Pologne. 1968;38:275–328.

[30] Nowak W. *Stomiosphaerina* nov. gen. (incertae seclis) of the Upper Cretaceous in the Polish Flysch Carpathians. Ann la Soc Geol Pologne. 1974;44:51–63.

[31] Borza K. Die Gattung *Stomiosphaera* Wanner, 1940 in den Westkarpathen. Geol Zborník–Geol Carpathica. 1964;15:189–195.

[32] Borza K. Die Mikrofazies und Mikrofossilien des Oberjuras und der Unterkreide der Klippenzone der Westkarpaten. Bratislava: Slovenská Akademia Vied; 1969. 1–301p.

[33] Borza K. *Carpistomiosphaera valanginiana* n. sp. and *Colomisphaera lucida* n. sp. from the Lower Cretaceous of the West Carpathians. Geol Zborník–Geol Carpathica. 1986;37(1):17–34.

[34] Řehánek J. Berriasian *Stomiosphaerina proxima* n. sp. (Stomiosphaeridae) from the Central West Carpathian Paleogene basal breccias. Geol Zborník–Geol Carpathica. 1987;38(6):695–703.

[35] Reháková D, Michalík J. Abundance and distribution ofLate Jurassic-Early Cretaceous microplankton in Western Carpathians. Geobios. 1994;27(2):135–156.

[36] Reháková D, Michalík J. *Stomiosphaera* or *Orthopithonella*? *Cadosina* or *Obliquipithonella*? notes to ultrastructure and syst ema tic pos it ion of some Jurassic–Cretaceous dinoflagellates from Western Carpathians. Miner Slovaca. 1996;28:92–98.

[37] Lintnerová O, Michalík J, Reháková D, Peterčáková M, Halásová E, Hladíková J. Sedimentary and isotopic record of the Aptian anoxic Selli event in the Pieniny Klippen Belt, Slovakia. Miner Slovaca. 1997;29:315–316.

[38] Michalík J, Reháková D, Lintnerová O, Boorová D, Halásová E, Kotulová J. Sedimentary, biological and isotopic record of early Aptian paleoclimatic event in the Pieniny Klippen Belt, Slovak Western Carpathians. Geol Carpathica. 1999;50(2): 169–191

[39] Michalík J, Reháková D, Halásová E, Lintnerová O. The Brodnosection–a potential regional stratotype of the Jurassic/Cretaceous boundary (Western Carpathians). Geol Carpathica. 2009;60:213–232.

[40] Michalík J, Reháková D, Grabowski J, Lintnerová O, Svobodová A, Schlögl J, et al. Stratigraphy, plankton communities, and magnetic proxies at the Jurassic/Cretaceous boundary in the Pieniny Klippen Belt (Western Carpathians, Slovakia). Geol Carpathica. 2016;67(4):303–328.

[41] Reháková D. Evolution and distribution of the Late Jurassic and Early Cretaceous calcareous dinofagellates recorded in the Western Carpathians pelagic carbonate facies. Miner Slovaca. 2000;32:79–88.

[42] Reháková D. Calcareous dinoflagellate and calpionellid bioevents versus sea-level fluctuations recorded in the West-Carpathian (Late Jurassic/Early Cretaceous) pelagic environments. Geol Carpathica. 2000;51(4):229–243.

[43] Pszczółkowski A, Myczyński R. Ammonite supported microfossil and nannoconid stratigraphy of the Tithonian–Hauterivian limestones in selected sections of the Branisko Succession, Pieniny Klippen Belt (Poland). Stud Geol Pol. 2004;123:133–197.

[44] Olszewska B. Microfossils of the Cieszyn Beds (Silesian Unit, Polish Outer Carpathians)–a thin sections study. Polish Geol Inst Spec Pap. 2005;19:1–58.

[45] Olszewska B, Szydło A, Jugowiec-Nazarkiewicz M, Nescieruk P. Integrated biostratigraphy of carbonate deposits of Cieszyn Beds in the Polish Western Carpathians. Geologia. 2008;34:33–59.

[46] Pszczółkowski A, Grabowski J, Wilamowski A. Integrated biostratigraphy and carbon isotope stratigraphy of the Upper Jurassic shallow water carbonates of the High-Tatric Unit (Mały Giewont area, Western Tatra Mountains, Poland). Geol Q. 2016;60(4):893–918.

[47] Ciurej A, Bąk K, Bąk M. Late Albian calcareous dinocysts and calcitarchs record linked to environmental changes during the final phase of OAE 1d –a case study from the Tatra Mountains, Central Western Carpathians. Geol Q. 2017;61(4):95–103.

[48] Meier KJS, Engemann N, Gottschling M, Kohring R. Die Bedeutung der Struktur der Zystenwand kalkiger Dinoflagellaten (Thoracosphaeraceae, Dinophyceae). Berliner Palobiologische Abhandlungen. 2009;10:245–256.

[49] Scheibner E. *Cadosinopsis*, nouveau genre du Crétacé supérieur de lazone des Klippes (Carpathes occidentales). Rev Micropaléontologie. 1967;10(1):42–47.

[50] Borza K. Cadosinopsis nowaki n. sp. (incerte sedis) from the Lower Cretaceous of the West Carpatians. Geol Zborník–Geol Carpathica. 1984;35:649–661.

[51] Skupien P, Doupovcová P. Dinoflagellates and calpionellids of the Jurassic–Cretaceous boundary, Outer Western Carpathians (Czech Republic). Cretac Res. 2019;99:209–228.

[52] Ivanova D. *Cadosinopsis nowaki*—a new calcareous dinocyst zone (Upper Valanginian-Lower Hauterivian) in West Bulgaria. Comptes rendus l’Académie Bulg des Sci Sci mathématiques Nat. 2001;54(1):55–58.

[53] Birkenmajer K. Jurassic and Cretaceous lithostratigraphic units of the Pieniny Klippen Belt, Carpathinas. Stud Geol Pol. 1977;45:1–159.

[54] Prokešová R, Plašienka D. and Milovský R. Structural pattern and emplacement mechanisms of the Krížna cover nappe (Central Western Carpathians). Geologica Carpathica 2012;63,13–32. http://www.geologicacarpathica.com/browse-journal/volumes/63-1/article-582/.

[55] Watycha J. Detailed Geological Map of Poland, scale 1:50,000; Czarny Dunajec sheet (1048). Państwowy Instytut Geologiczny, Warszawa,1974. [in Polish] http://bazadata.pgi.gov.pl/data/smgp/arkusze_skany/smgp1048.jpg.

[56] Watycha J. Explanations to the Detailed Geological Map of Poland, scale 1:50,000; Czarny Dunajec sheet (1048). Re-edited by Gaździcka E., Żarski M. and Sważdba R. (2019). Państwowy Instytut Geologiczny-Państwowy Instytut Badawczy, Warszawa, 1974. 74 pp. and 4 plates [in Polish].

[57] Bąk M, Chodacka S, Bąk K, Okoński S. New data on the age and stratigraphic relationships of the Czajakowa Radiolarite Formation in the Pieniny Klippen Belt (Carpathians) based on the radiolarian biostratigraphy in the stratotype section. Acta Geol Pol. 2018;68(1):1–20.

[58] Ryan PD, Harper DAT, Whalley JS. PALSTAT: User’s Manual and Case Histories: Statistics for palaeontologists and palaeobiologists. Chichester: Chapman & Hall; 1995. 1–73 p.

[59] Harper DAT. Numerical Palaeobiology: Computer-based Modelling and Analysis of Fossils and their Distributions. Portland: John Wiley & Son; 1999. 1–478 p.

[60] Hammer Ø, Harper DAT, Ryan PD. Past: Paleontological statistics software package for education and data analysis. Palaeontol Electron. 2001;4(1):1–9.

[61] Řehánek J. *Cadosinidae* Wanner and *Stomiosphaeridae* Wanner (incertae sedis) from the Mesozoic limestone of southern Moravia. Časopis pro Mineral a Geol. 1985;30:367–378.

[62] Ivanova D, Keupp H. Calcareous dinoflagellate cysts from the Late Jurassic and Early Cretaceous of the Western Forebalkan. Berliner Geowissenschaftliche Abhandlungen, 1999; 30:3–31.

